# Effect of Angle on Flow-Induced Vibrations of Pinniped Vibrissae

**DOI:** 10.1371/journal.pone.0069872

**Published:** 2013-07-26

**Authors:** Christin T. Murphy, William C. Eberhardt, Benton H. Calhoun, Kenneth A. Mann, David A. Mann

**Affiliations:** 1 College of Marine Science, University of South Florida, St. Petersburg, Florida, United States of America; 2 Department of Mechanical and Aerospace Engineering, University of Virginia, Charlottesville, Virginia, United States of America; 3 Electrical and Computer Engineering, University of Virginia, Charlottesville, Virginia, United States of America; 4 Department of Orthopedic Surgery, SUNY Upstate Medical University, Syracuse, New York, United States of America; Instituto de Neurociencias de Alicante UMH-CSIC, Spain

## Abstract

Two types of vibrissal surface structures, undulated and smooth, exist among pinnipeds. Most Phocidae have vibrissae with undulated surfaces, while Otariidae, Odobenidae, and a few phocid species possess vibrissae with smooth surfaces. Variations in cross-sectional profile and orientation of the vibrissae also exist between pinniped species. These factors may influence the way that the vibrissae behave when exposed to water flow. This study investigated the effect that vibrissal surface structure and orientation have on flow-induced vibrations of pinniped vibrissae. Laser vibrometry was used to record vibrations along the whisker shaft from the undulated vibrissae of harbor seals (*Phoca vitulina*) and northern elephant seals (*Mirounga angustirostris*) and the smooth vibrissae of California sea lions (*Zalophus californianus*). Vibrations along the whisker shaft were measured in a flume tank, at three orientations (0°, 45°, 90°) to the water flow. The results show that vibration frequency and velocity ranges were similar for both undulated and smooth vibrissae. Angle of orientation, rather than surface structure, had the greatest effect on flow-induced vibrations. Vibration velocity was up to 60 times higher when the wide, flat aspect of the whisker faced into the flow (90°), compared to when the thin edge faced into the flow (0°). Vibration frequency was also dependent on angle of orientation. Peak frequencies were measured up to 270 Hz and were highest at the 0*°* orientation for all whiskers. Furthermore, CT scanning was used to quantify the three-dimensional structure of pinniped vibrissae that may influence flow interactions. The CT data provide evidence that all vibrissae are flattened in cross-section to some extent and that differences exist in the orientation of this profile with respect to the major curvature of the hair shaft. These data support the hypothesis that a compressed cross-sectional profile may play a key role in reducing self-noise of the vibrissae.

## Introduction

Vibrissae, otherwise known as sinus hairs, sensory hairs, tactile hairs, or whiskers, are keratinous structures of epidermal origin that are present in nearly all mammals [1]. These structures are especially well developed in pinnipeds (seals, sea lions, and walrus). The vibrissae are arranged in an array about the face and muzzle and connect to richly innervated follicle sinus complexes below the skin [2], [3], [4]. In pinnipeds, the vibrissae are known to function in both haptic touch and detection of waterborne disturbances from hydrodynamic stimuli [5], [6], [7], [8]. The vibrissal system has been demonstrated to be highly sensitive and, in some seals, has been shown to detect water velocities as low as 245 µms^−1^ [6].

**Figure 1:**
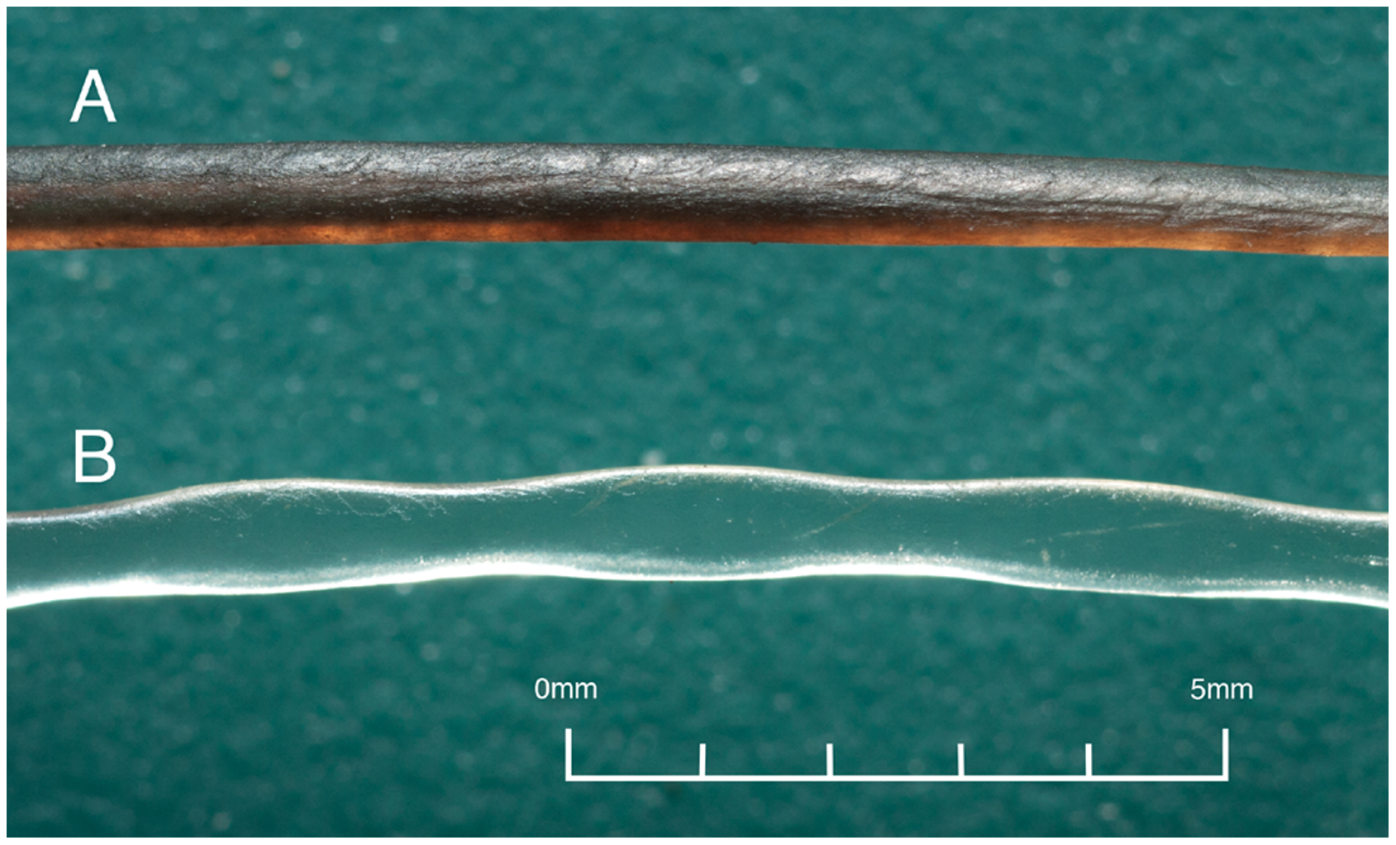
Undulated and smooth vibrissal surface structures. Surface structure of (A) a smooth vibrissa (California sea lion) and (B) an undulated vibrissa (harbor seal).

Two types of vibrissal surface structures, undulated and smooth, exist among pinnipeds. The vibrissae of all Phocidae (true seals), with the exception of the bearded seal (*Erignathus barbatus)* and monk seals (*Monachus spp.)*, have undulated surfaces. This surface profile has also been described as wavy, beaded, or corrugated in appearance and is characterized by repeating crests and troughs along the length of the shaft [5], [9]. In contrast, the vibrissae of all Otariidae (fur seals and sea lions) and Odobenidae (walrus) have smooth surfaces (Figure 1).

Pinnipeds are the only animal group known to possess undulated vibrissae [10]. The unique morphological differences in pinniped vibrissal surface structure have been noted by numerous investigators [2], [5], [9], [11], [12], but their functional relevance is unclear. It has been hypothesized that the undulated structure facilitates detection of hydrodynamic signals, possibly by enhancing sensitivity or reducing background noise on the sensor [7], [8], [10], [11], [13]. Hanke et al. [14] and Miersch et al. [15] recently reported experimental evidence suggesting that the undulations serve to minimize vortex shedding behind the whisker, thereby reducing vibrations that would be generated from movement through the water. The experiments utilized force measurements and computational fluid dynamics to compare the flow resistance between undulated and smooth vibrissae. Using piezoceramic transducers, the dynamic forces at the base of the shaft were compared between the undulated vibrissae of harbor seals and the smooth vibrissae of California sea lions, measured in a rotational flume with the hairs at one fixed angle relative to the flow. The studies revealed forces up to 9.5 times lower at the base of the undulated vibrissae than at the base of the smooth vibrissae, as well as a lower signal to noise ratio on the harbor seal than the sea lion vibrissae. A subsequent study [16], which combined particle imaging techniques and numerical simulation, determined that the wake behind an undulated vibrissa is characterized by an unsteady vortex structure and resulted in reduced drag and lift forces, as compared to an infinite cylinder with a circular cross-section.

While previous experimental evidence [14] suggests that undulated vibrissae may be more specialized for hydrodynamic detection, behavioral studies indicate that both species with smooth vibrissae and species with undulated vibrissae can effectively track hydrodynamic signals [7], [8]. In addition, psychophysical testing has revealed that pinnipeds with either type of vibrissae can detect low amplitude waterborne vibrations. At certain frequencies, the smooth vibrissae of the California sea lion actually have better sensitivity than the undulated vibrissae of the harbor seal [17], suggesting that surface structure alone may not be the only factor influencing performance.

In addition to differences in surface structure, it is also important to consider that cross-sectional shape of the hair shaft may affect the behavior of vibrissae when exposed to water flow. While all terrestrial mammals have smooth vibrissae with a circular cross-section [10], both undulated and smooth pinniped vibrissae are flattened in profile to some extent [2], [14], [18]. It has been hypothesized that undulated vibrissae exhibit more extreme flattening, while the smooth vibrissae have an oval cross-section [17]. In addition, there may be variability between species in the degree of cross-sectional flattening; however, this has not been studied in detail. Due to the cross-sectional flattening that occurs in both whisker types, each vibrissa has a distinct broad and thin aspect. Slightly rotating the vibrissa will change which edge faces into the flow, thus making orientation of the vibrissa a potentially important consideration in understanding flow interactions.

In addition to structural differences, variations in positioning and orientation of the array may exist between pinniped groups. Pinnipeds have motor control over the vibrissae and can protract the sensors from a relaxed position, flat against the face, to an erect position, held nearly perpendicular to the axis of the body. Previous behavioral studies of hydrodynamic wake following indicate that both harbor seals and California sea lions hold the vibrissal array in the erect position while tracking signals underwater [7], [8]. It is not currently understood how the vibrissae are oriented in awake, behaving animals and how this may differ between species. However, it is important to consider these factors as a biological framework for understanding flow interactions of vibrissae.

Previous researchers have demonstrated that water flow causes vibrations along the vibrissal shaft [15], [19]. A recent study conducted in air also found that seal vibrissae vibrated in response to stimulation from low frequency sounds [20]. When modeled with the appropriate drag coefficient for water, the vibrissae were predicted to be tuned to frequencies of 20 to 200 Hz. In addition, some prior data demonstrated that orientation of the vibrissae influences the vibrations elicited by movement through the water. Hyvärinen measured the frequency of vibrations along the shaft of a single excised vibrissa of the Saimaa ringed seal and found that when held with the broad edge of the vibrissa facing into the flow, vibrations along the shaft were measured at up to 300 Hz [21]. However, when the same vibrissa was held with the narrow edge of the hair shaft facing into the flow, no detectable vibrations were measured.

The goal of the present study was to measure the effect that orientation has on the vibrations of smooth and undulated pinniped vibrissae exposed to water flow in a flume tank. Vibrissae were tested from three species of pinnipeds, one with smooth vibrissae and two with undulated vibrissae. Samples were fixed at multiple angles to the flow and the velocity and frequency spectra of vibrations were analyzed with respect to orientation. In addition, CT scanning of vibrissal samples was conducted in order to quantify the cross-sectional shape of the vibrissae and understand how this may contribute to orientation effects on self-induced vibrations.

## Materials and Methods

### Sample Collection

Testing was conducted on excised mystacial vibrissae samples collected from post-mortem stranded animals at the Marine Mammal Center in Sausalito, California. Samples were collected from three pinniped species: the Pacific harbor seal (*Phoca vitulina*), northern elephant seal (*Mirounga angustirostris*), and California sea lion (*Zalophus californianus*). Sample availability was restricted to juvenile animals due to the higher mortality rate of this age group and both male and female animals were sampled. All samples were collected from the right side of the muzzle and one vibrissa from each individual was tested.

Vibrissal samples for flume testing were collected from 26 individuals (n = 9 for California sea lions; n = 8 for elephant seals; n = 9 for harbor seals). In order to standardize size across samples, vibrissae of matching lengths were selected for testing. Mean length of 7.7 cm (s.d. = 0.55 cm) was chosen because samples in this size class were present in all three species. Constraining sample length was prioritized over matching for follicle position on the vibrissal bed. Length was constrained as closely as sample availability would allow. Mean length by species was 7.7 cm (s.d. = 0.4 cm) for California sea lions; 8.19 cm (s.d. = 0.5 cm) for elephant seals; and 7.35 cm (s.d. = 0.4 cm) for harbor seals.

During collection, vibrissae were clipped at the skin surface, rinsed in fresh water, and packaged in dry gauze for transport. Prior to testing, each specimen was rehydrated by immersion in fresh water for one hour. Rehydration as well as flume testing was conducted in fresh water due to constraints of the flume setup.

Vibrissal samples from an additional 9 individuals (n = 3 for California sea lions; n = 3 for elephant seals; n = 3 for harbor seals) were collected for CT scanning in order to quantify the three-dimensional shapes of the vibrissae. Samples for this portion of the study were also selected based on the length criteria used in flume testing. Mean length for CT samples was 6.5 cm (s.d. = 0.63 cm). All samples were collected from the right side of the muzzle, and one vibrissa from each individual was scanned. These samples were extracted from the capsule, leaving the subdermal portion of the hair shaft attached. Samples were rehydrated in fresh water prior to being mounted on a slide for CT scanning.

### Flume Apparatus and Vibration Recordings

**Figure 2:**
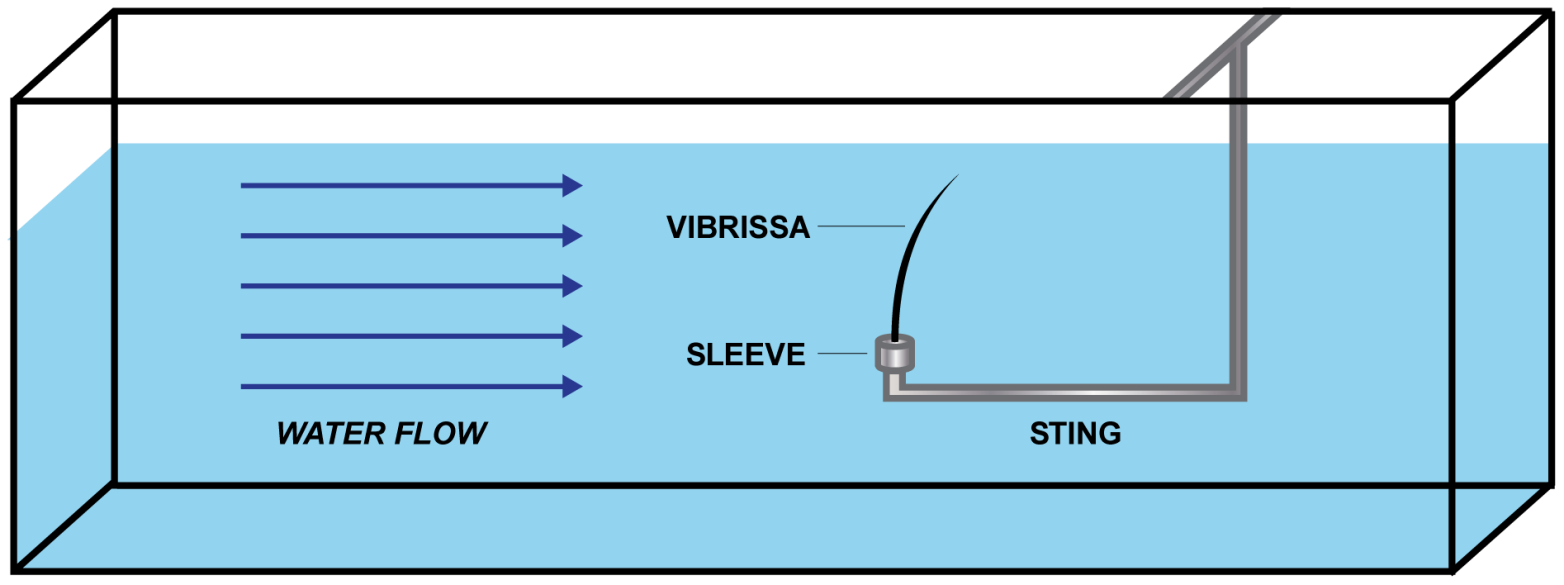
Diagram of a vibrissal sample mounted in the test section of the water flume. Schematic (figure not drawn to scale) of the recording area of the flume. The vibrissa was mounted on the sting apparatus in the center of the water column. The laser vibrometer (not pictured) was focused on the vibrissal shaft from outside the test enclosure, with the beam passing through the water column, perpendicular to the flow.

Samples were tested in a Rolling Hills Research Company Model 1520 water flume (El Segundo, CA, USA). The test section of the flume measures 152 cm in length, 38 cm in width, and 46 cm tall. Flow in the test section was laminar with velocity uniformity outside the wall boundary layer of <+/−2%. Inside the flume, individual vibrissae were mounted on a sting apparatus composed of a stainless steel rod with a 90 degree bend (Figure 2), located in the center of the water column. The boundary layer thickness along the walls of the flume tank was 1.6 cm and did not extend into the location of the sample mount. The body of the sting mount was positioned downstream of the sample; therefore, it did not interfere with the flow around the whisker. For attachment to the sting, the sample was fixed inside a cylindrical, threaded aluminum sleeve. The base of each whisker was inserted 1 cm into the sleeve and set with epoxy. The threaded sleeve base allowed the whisker to be rotated on the sting in order to test various angles to the flow. All tests were conducted at a flow speed of 0.5 m/s, verified by particle image analysis (PIV). The flow speed was selected based on the constraints of the system, as higher flow rates caused secondary vibrations of the tank that would have affected the data collected from the vibrissae. Although 0.5 m/s represents a slow swimming speed for these animals, it is represented in the swimming behavior of the species studied and may be more typical of glide phases in swimming [22], [23], [24], [25], [26].

**Figure 3:**
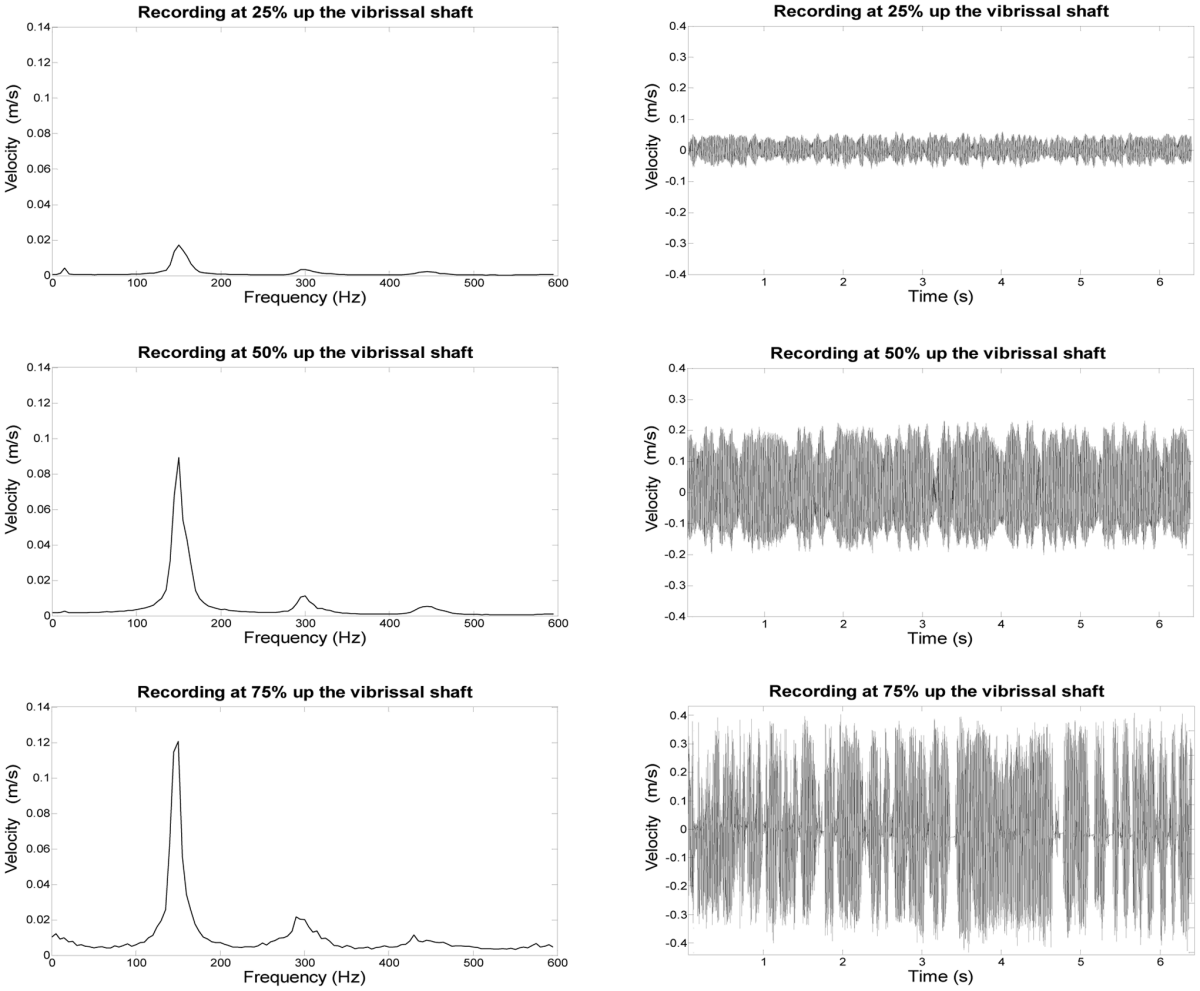
Vibrational signal with distance up the vibrissal shaft. Comparison of three example laser vibrometer recordings taken at different points along the shaft of a single vibrissal sample. Recordings were taken at 25% (top row), 50% (middle row), and 75% (bottom row) up the length of the whisker shaft and are shown as FFTs (left) and waveforms (right). The 50% recording position was determined to be optimal for signal quality and all subsequent data were recorded at this position.

Recordings were made with a Polytec model PDV 100 laser-Doppler vibrometer, measuring point velocities on the whisker (Waldbronn, Germany). The laser was focused on the whisker shaft and vibrations in the cross-stream direction were recorded for 6 seconds using the Polytec Vibrometer Software (version 4.6). Pilot recordings were conducted to determine the optimal location along the whisker shaft to record vibrations. Vibration frequency remained consistent along the whisker length, while velocity increased with distance from the base. Vibration velocity was maximal at the distal end of the vibrissa. However, recording quality degraded at the tip because the large whisker displacement sometimes moved it out of the plane of the laser (Figure 3). All comparative recordings were therefore collected at the midpoint of the whisker’s length, where the recording equipment collected a strong, consistent signal.

**Figure 4:**
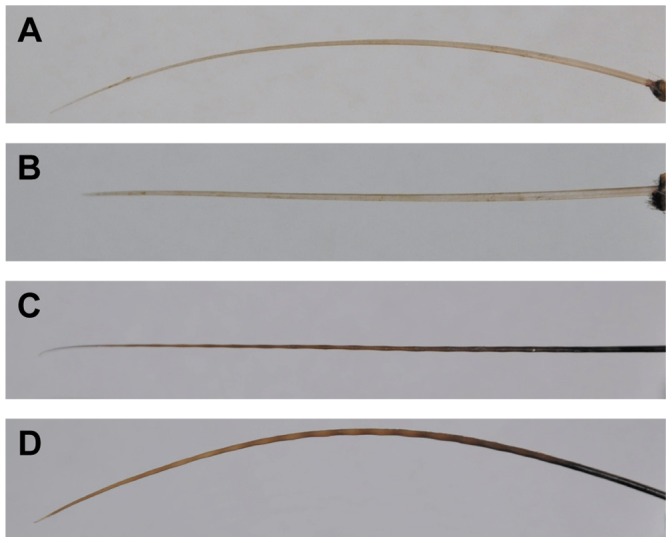
Vibrissal orientation for laser vibrometer recordings. (A) Smooth (California sea lion) vibrissa at the 0° orientation. Thin edge of the vibrissa faces into the flow. (B) The same vibrissa at the 90° orientation. Broad edge of the vibrissa faces into the flow. (C) Undulated vibrissa (elephant seal) at the 0° orientation. Thin edge of the vibrissa faces into the flow. (D) The same vibrissa at the 90° orientation. Broad edge of the vibrissa faces into the flow. In these images, the direction of flow is into the page. Total length of the vibrissa in A and B is 8.1 cm, total length of the vibrissa in C and D is 9.2 cm.

**Figure 5:**
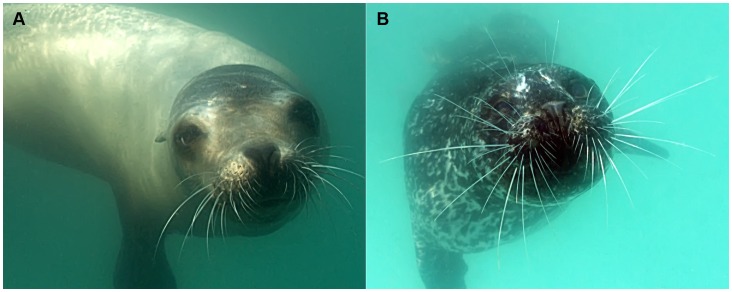
Position of the vibrissal array during active swimming. (A) California sea lion with the vibrissal array protracted. In this position the vibrissae are curved ventrally. (B) Harbor seal with the vibrissal array protracted. In this position the vibrissae are curved caudally.

Each sample was tested at vibrissae orientations of 0°, 45°, and 90° to the flow. Vibration measurements were recorded in the cross-stream direction for all vibrissae orientations. Definition of orientation was based on the angle of attack, or angle of the major cross-sectional axis of the vibrissa, at the base of the sample. Caliper measurements were taken at the base of the sample to determine the position of the minimum caliper width. This position was defined as 0° and the thin edge of the base of the vibrissa faced into the flow at this orientation. As the sample was rotated to 45° and then 90°, the broad edge of the vibrissa was oriented into the flow. In the 0° condition, the major curvature of the hair shaft was generally in the downstream direction for the harbor and elephant seal samples and in the cross-stream direction for the California sea lion samples (Figure 4). Based on behavioral observations, we hypothesize that the 0° condition closely corresponds to the natural orientation of the vibrissae in each species when the array is protracted (Figure 5).

Signals from the laser vibrometer were digitized at 1200 Hz and signal processing was conducted in MATLAB (Mathworks, Inc.). Peak frequency and corresponding peak vibration velocity were determined by performing fast Fourier transform (FFT) averaging with a 240 point FFT, yielding a frequency resolution of 5 Hz. Analysis was conducted on the entire 6 second length for most recordings. For some recordings that showed sections of reduced signal quality, shorter segments were analyzed with fewer power spectra averaged. One sea lion recording and three harbor seal recordings were removed from the analysis due to large vibrations in the stream-wise direction causing the whisker to move out of line with the laser taking measurements in the cross-stream direction.

A linear regression analysis was performed in order to analyze the variation in frequency and velocity that was attributable to length of the vibrissal sample. Repeated-measures two-factor ANOVA analyses with Tukey post-hoc tests were conducted to examine the effect of species and angle of orientation on frequency and velocity of whisker vibration (GraphPad Prism Software version 6, San Diego, CA). As statistical tests did not allow for uneven sample size within repeated measures analyses, subjects that had dropped values at the 90° orientation due to poor laser vibrometer signal were removed from the analysis. The resulting sample size for statistical tests was n = 8 for California sea lions; n = 8 for elephant seals; and n = 6 for harbor seals.

### Calculated Theoretical Vibration Frequency

The recorded whisker vibration frequency was compared to calculated theoretical vortex shedding frequency for a cylinder of similar size, with a circular cross-section. The theoretical cylinder used for modeling was based on the diameter of the vibrissa facing into the flow at the corresponding angle of orientation. Theoretical cylinders modeled only the stream-wise diameters of the whiskers and not the surface structure. In addition, calculations modeled only frequency and not velocity of vibrations.

The theoretical vibration frequency is based on the Reynolds number calculation:
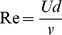


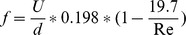
where Re = Reynolds number, U = fluid velocity, d = vibrissae cross-sectional length perpendicular to the flow, ν = kinematic viscosity, f = frequency [27], [28].

Theoretical frequency was calculated for each vibrissa at the 0° and 90° orientations based on the width of the profile facing into the flow at each orientation. These stream-wise diameters were measured at the centroid of the vibrissa. For undulated vibrissae, a maximum and minimum width was measured at points closest to the centroid, due to the variations in thickness of the shaft created by the crests and troughs. The 45° orientation was omitted from these calculations because there was potential for error in the measurement of the stream-wise diameter, due to the fact that the profile facing into the flow was an angled plane.

### CT Scanning of Vibrissal Structure

Samples were rehydrated in fresh water for one hour and taped flat onto an acrylic slide, without compromising the natural curvature of the hair shaft. Samples were scanned in air using a micro-CT scanner (SCANCO, Wayne, NJ) at 30 µm isotropic resolution. Three dimensional reconstructions and digital cross-section images were created in ImageJ (version 1.47d) [29]. Cross-sectional properties (cross-sectional area, maximum and minimum caliper width, and theta or the angle of the principal cross-sectional axis) were determined using BoneJ (version1.3.7) [30]. Based on the maximum and minimum chord dimensions, eccentricity or ellipticity (a measure of how much an ellipse deviates from being circular) was calculated for each cross-sectional slice. Eccentricity *(e)* is based on the equation:
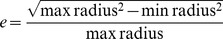
with the eccentricity of a circle being 0 and the eccentricity of an ellipse being >0 but <1.

### Underwater Photographic Images

Images of the vibrissae in a live, free-swimming harbor seal and California sea lion were captured at Long Marine Laboratory at the University of California Santa Cruz, using the Phantom Flex high speed underwater video camera (Vision Research Inc., Wayne, NJ). Images were recorded at 400 to 1000 frames per second and still shots were isolated using the Vision Research PCC software.

### Ethics Statement

The use of marine mammal samples was authorized under the National Marine Fisheries Service, letter of authorization to C. Murphy. Research with live marine mammals was authorized under National Marine Fisheries Service permit 14535 and conducted with the approval of the Institutional Animal Care and Use Committee at the University of California Santa Cruz.

## Results

**Figure 6:**
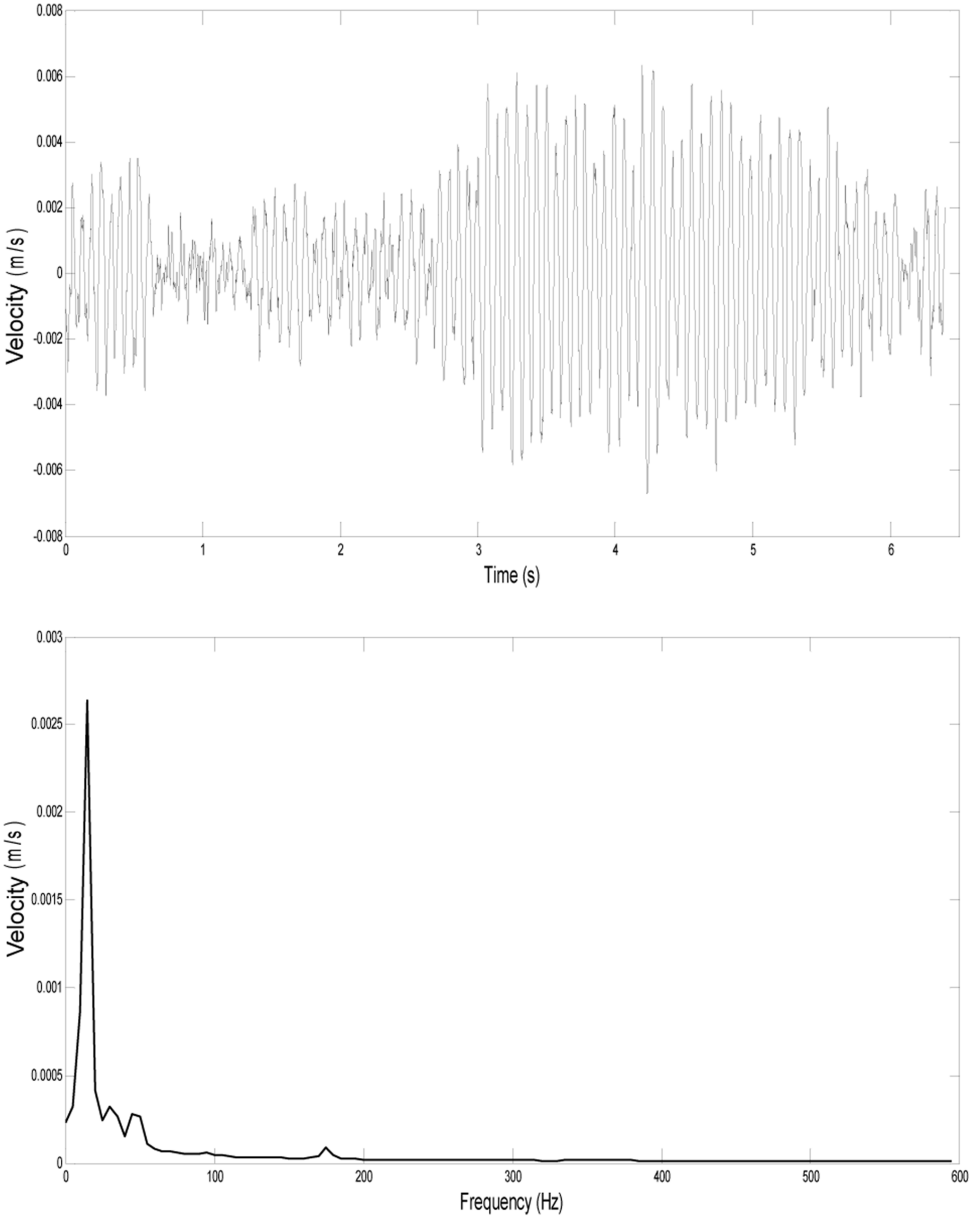
Vibrational signal recorded from the sting mount. Vibration of the sting apparatus, shown as a waveform (top) and FFT (bottom). The peak frequency of the vibration of the sting apparatus was consistently at 15 Hz and did not overlap with the frequency range of the signal from the vibrissae. Note that the scale used here to view the sting vibration is approximately 50 times smaller than the scales used for the vibrissae vibrations in Figures 3 and 7.

Whiskers vibrated strongly with a distinct fundamental frequency. Harmonics were observed in some recordings but were not consistently seen for all samples. The vibration of the sting apparatus, on which the sample was mounted, was recorded and did not overlap with the frequency range of the vibrissae vibration (Figure 6). The sting apparatus consistently vibrated under 50 Hz, with a peak frequency of 15 Hz, and peak velocity less than .005 m/s.

**Figure 7:**
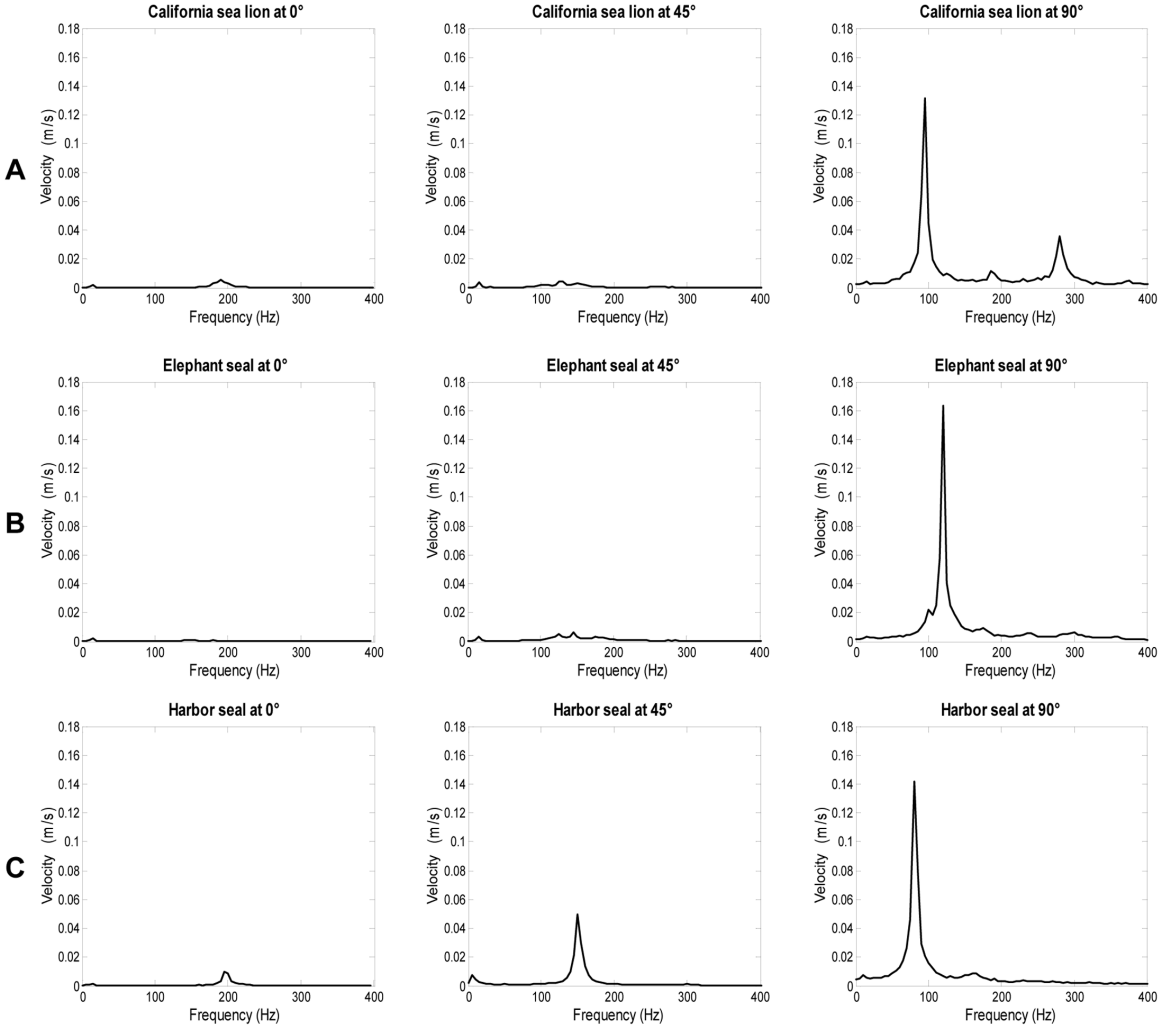
Effect of angle of orientation on vibrational signal. Comparison of FFTs at 0°, 45°, and 90° for one individual (A) California sea lion, (B) elephant seal and (C) harbor seal. In all vibrissae, peak velocity was minimal at the 0° orientation and increased as the vibrissa was rotated to 45° and then 90°.

**Figure 8:**
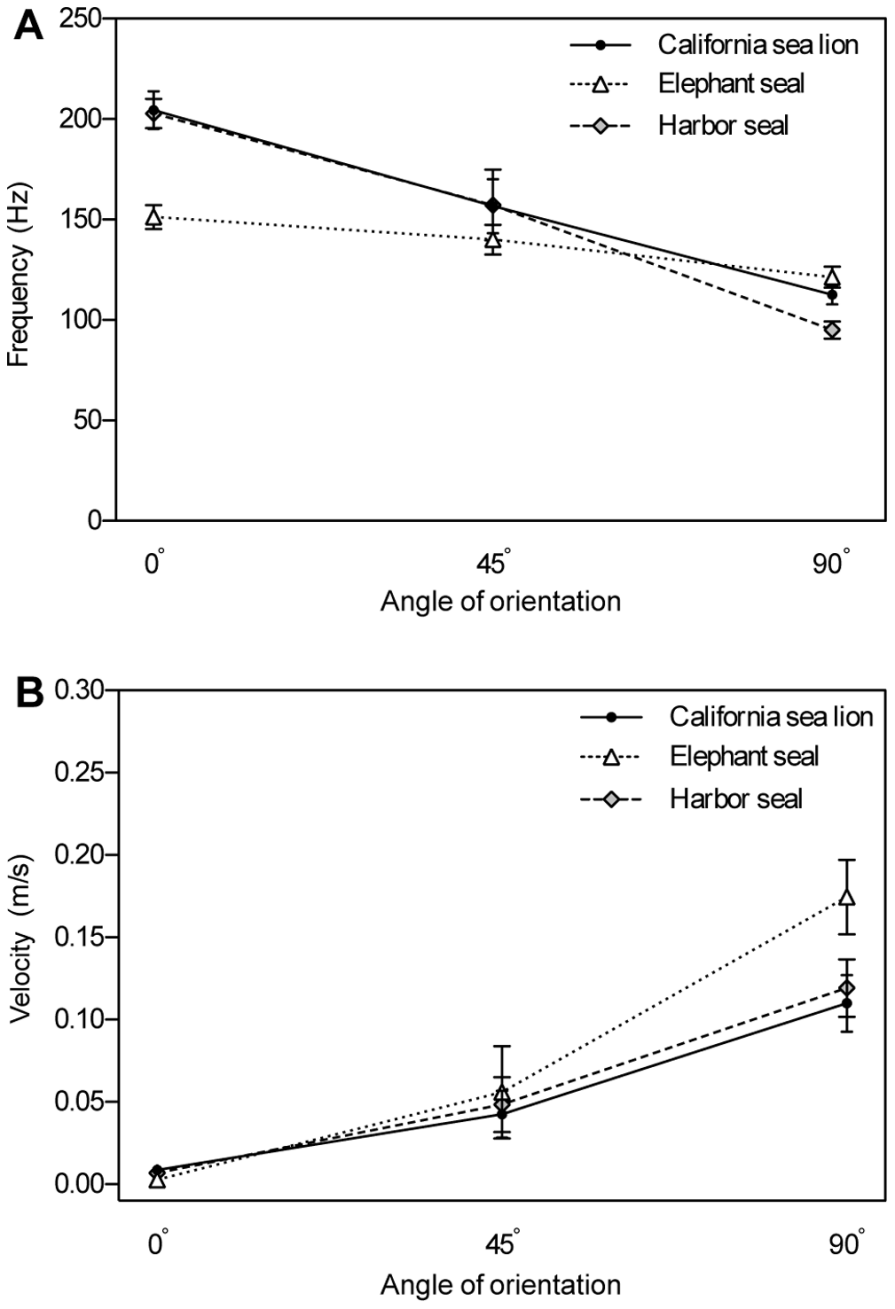
Effect of angle of orientation on mean peak frequency and velocity of vibration. (A) Mean peak frequency across species at three angles of orientation. For all whisker types, peak frequency was highest at the 0° orientation and decreased as the vibrissa was rotated to 45° and then 90°. (B) Mean peak velocity across species at three angles of orientation. In all vibrissae, peak velocity was lowest at the 0° orientation and increased as the vibrissa was rotated to 45° and then 90°. Both graphs show pooled data for each species with +/− SE.

Vibration frequency and velocity ranges were similar for both undulated and smooth vibrissae (Table 1 and Table 2). At each angle of orientation, similar values were observed across whisker types. Angle of orientation affected the peak frequency and peak velocity of vibrations. For all whisker types, peak frequency was highest at the 0° orientation and decreased as the vibrissa was rotated to 45° and then 90° (Figure 7, Figure 8A). Theoretical frequency, calculated based on Reynolds number for a cylinder of similar size, (Table 1) revealed the same trend in values. The theoretical frequency calculations based on stream-wise diameter alone yielded average predicted values that were within 21% of the corresponding measured value. For all whisker types, peak velocity was minimal at the 0° orientation and increased as the vibrissa was rotated to 45° and then 90° (Figure 7, Figure 8B).

**Table 1:** Measured and theoretical vibration frequency values pooled across subjects for each species group.

		Measured Peak Frequency (Hz)			Theoretical Frequency (Hz)			
	Angle of orientation	0°	45°	90°	0°	0° %difference	90°	90° % difference
**California sea lion**	**Mean**	**204.4**	**156.7**	**112.5**	**201.1**	4.9%	**122.2**	14.6%
(Smooth vibrissae)	S.E.	9.4	13.4	4.6	8.7		5.3	
	Range	175.0–270.0	115.0–230.0	95.0–130.0	159.0–233.4		98.9–137.1	
**Elephant seal**	**Mean**	**151.3**	**140.0**	**121.3**	**176.3**	18.3%	**102.6**	14.8%
(Undulated vibrissae)	S.E.	5.9	7.3	5.2	5.7		2.0	
	Range	130.0–185.0	115.0–170.0	105.0–140.0	154.5–201.9		94.5–111.2	
**Harbor seal**	**Mean**	**202.8**	**157.2**	**95.0**	**241.8**	20.9%	**103.4**	9.4%
(Undulated vibrissae)	S.E.	7.3	17.6	4.3	7.9		2.8	
	Range	175.0–240.0	70.0–245.0	80.0–110.0	195.5–271.9		93.4–112.7	

Overall range, mean, and SE of the measured peak frequency and calculated theoretical frequency for all vibrissae sampled. Measured values were obtained from laser vibrometer recordings and theoretical values are based on the Reynolds number calculation for a cylinder with a circular cross-section and matching stream-wise diameter. The percent difference between the absolute value of the measured and theoretical frequency was calculated for each vibrissa. The “% difference” column is the mean of the percent differences pooled across subjects for the specified species and angle of orientation. Samples that had dropped values at the 90° orientation due to poor laser vibrometer signal were removed from the data set. The resulting sample size was n = 9 at 0°, n = 9 at 45°, and n = 8 at 90° for California sea lions; n = 8 at 0°, n = 8 at 45°, and n = 8 at 90° for elephant seals; and n = 9 at 0°, n = 9 at 45°, and n = 6 at 90° for harbor seals.

**Table 2:** Measured vibration velocity values pooled across subjects for each species group.

		Measured Peak Velocity (m/s)		
	Angle oforientation	0°	45°	90°
**California sea lion**	**Mean**	**0.0087**	**0.0426**	**0.1099**
(Smoothvibrissae)	S.E.	0.0029	0.0142	0.0172
	Range	0.0036–0.0316	0.0051–0.1452	0.0535–0.2018
**Elephant seal**	**Mean**	**0.0029**	**0.0559**	**0.1745**
(Undulated vibrissae)	S.E.	0.0010	0.0280	0.0226
	Range	0.0007–0.0091	0.0034–0.1842	0.0909–0.3083
**Harbor seal**	**Mean**	**0.0069**	**0.0484**	**0.1192**
(Undulated vibrissae)	S.E.	0.0038	0.0167	0.0175
	Range	0.0009–0.0361	0.0022–0.1481	0.0484–0.1745

Overall range, mean, and SE of the measured peak velocity from laser vibrometer recordings for all vibrissae sampled. Samples that had dropped values at the 90° orientation due to poor laser vibrometer signal were removed from the data set. The resulting sample size was n = 9 at 0°, n = 9 at 45°, and n = 8 at 90° for California sea lions; n = 8 at 0°, n = 8 at 45°, and n = 8 at 90° for elephant seals; and n = 9 at 0°, n = 9 at 45°, and n = 6 at 90° for harbor seals.

For frequency data, a repeated-measures two-factor ANOVA showed a significant effect of angle of orientation on frequency of whisker vibration (p<0.0001). However, the main effect analysis for species did not reach the criterion for statistical significance (p = 0.0582). There was a significant interaction between species and angle of orientation (p<0.0001) (Table 3). The results of Tukey post-hoc tests are shown in Table 4. A linear regression analysis showed that very little of the variation in frequency (*b = −*20.164 Hz/cm; r^2^ = 0.074) was explained by length.

**Table 3:** ANOVA table for vibration frequency.

Source	SS	DF	MS	F (DFn, DFd)	P value
Angle of orientation	62950	2	31475	F (2, 38) = 74.62	<0.0001
Species	6690	2	3345	F (2, 19) = 3.315	0.0582
Interaction	14293	4	3573	F (4, 38) = 8.472	<0.0001
Subjects (matching)	19173	19	1009	F (19, 38) = 2.392	0.0109
Residual	16028	38	421.8		

ANOVA table showing the results of the repeated-measures two-factor analysis of variance, demonstrating the effect of species and angle of orientation on frequency of whisker vibration. A significant effect of angle of orientation and a significant interaction is observed. No significant effect of species is observed. Sample size for statistical tests was n = 8 for California sea lions; n = 8 for elephant seals; and n = 6 for harbor seals at all angles of orientation.

**Table 4:** Tukey post-hoc analysis of the effect of angle of orientation on vibration frequency, within each species.

Group	Mean diff.	P-value
California sea lion (CSL)		
CSL 0° vs. 45°	45.63	0.0002
CSL 0° vs. 90°	93.13	<0.0001
CSL 45° vs. 90°	47.5	0.0001
Elephant seal (ES)		
ES 0° vs. 45°	11.25	0.5227
ES 0° vs. 90°	30	0.0157
ES 45° vs. 90°	18.75	0.1749
Harbor seal (HS)		
HS 0° vs. 45°	17.5	0.3137
HS 0° vs. 90°	101.7	<0.0001
HS 45° vs. 90°	84.17	<0.0001

“Mean diff.” is the mean difference between the first and second angle listed. “P Value” is the adjusted p-value.

For velocity data, a repeated-measures two-factor ANOVA showed a significant effect of angle of orientation (p<0.0001), but no significant effect of species (p = 0.1732), on the velocity of whisker vibration. There was no statistically significant interaction between the effect of species and angle of orientation (p = 0.1669) (Table 5). The results of Tukey post-hoc tests are shown in Table 6. A linear regression analysis showed that very little of the variation in velocity (*b = *0.0067 mm/s·cm; *r* ^2^ = 0.003) was explained by length.

**Table 5:** ANOVA table for vibration velocity.

Source	SS	DF	MS	F (DFn, DFd)	P value
Angle of orientation	0.1873	2	0.09363	F (2, 38) = 51.15	P<0.0001
Species	0.009278	2	0.004639	F (2, 19) = 1.925	P = 0.1732
Interaction	0.01255	4	0.003138	F (4, 38) = 1.715	P = 0.1669
Subjects (matching)	0.04578	19	0.00241	F (19, 38) = 1.316	P = 0.2298
Residual	0.06955	38	0.00183		

ANOVA table showing the results of the repeated-measures two-factor analysis of variance, demonstrating the effect of species and angle of orientation on velocity of whisker vibration. A significant effect of angle of orientation is observed. No significant effect of species or interaction is observed. Sample size for statistical tests was n = 8 for California sea lions; n = 8 for elephant seals; and n = 6 for harbor seals at all angles of orientation.

**Table 6:** Tukey post-hoc analysis of the effect of angle of orientation on vibration velocity, within each species.

Group	Mean diff.	P-value
California sea lion (CSL)		
CSL 0° vs. 45°	−0.03359	0.2707
CSL 0° vs. 90°	−0.1008	<0.0001
CSL 45° vs. 90°	−0.06722	0.0089
Elephant seal (ES)		
ES 0° vs. 45°	−0.05295	0.046
ES 0° vs. 90°	−0.1715	<0.0001
ES 45° vs. 90°	−0.1186	<0.0001
Harbor seal (HS)		
HS 0° vs. 45°	−0.01882	0.7283
HS 0° vs. 90°	−0.1101	0.0002
HS 45° vs. 90°	−0.09124	0.002

“Mean diff.” is the mean difference between the first and second angle listed. “P Value” is the adjusted p-value.

**Figure 9:**
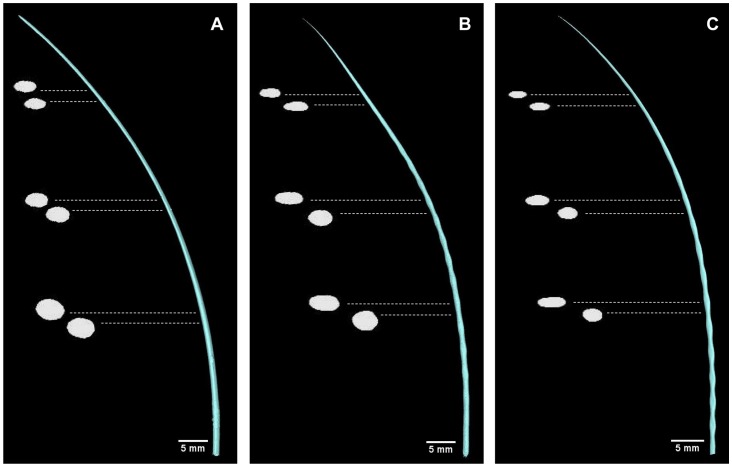
Comparative digital cross-sections from CT data. Reconstructions of vibrissae from CT scan data. (A) California sea lion; (B) elephant seal; (C) harbor seal. Enlarged digital cross-sections are shown at six points along the whisker length. Scale bar represents scaling for whole whisker image. Cross-sections are approximately 4–5x enlarged. In smooth vibrissae, the cross-sectional shape is consistent between neighboring points along the shaft, while undulated vibrissae vary in cross-sectional shape between troughs to crests. The cross-sections of all vibrissae show increased flattening toward the tip.

**Figure 10:**
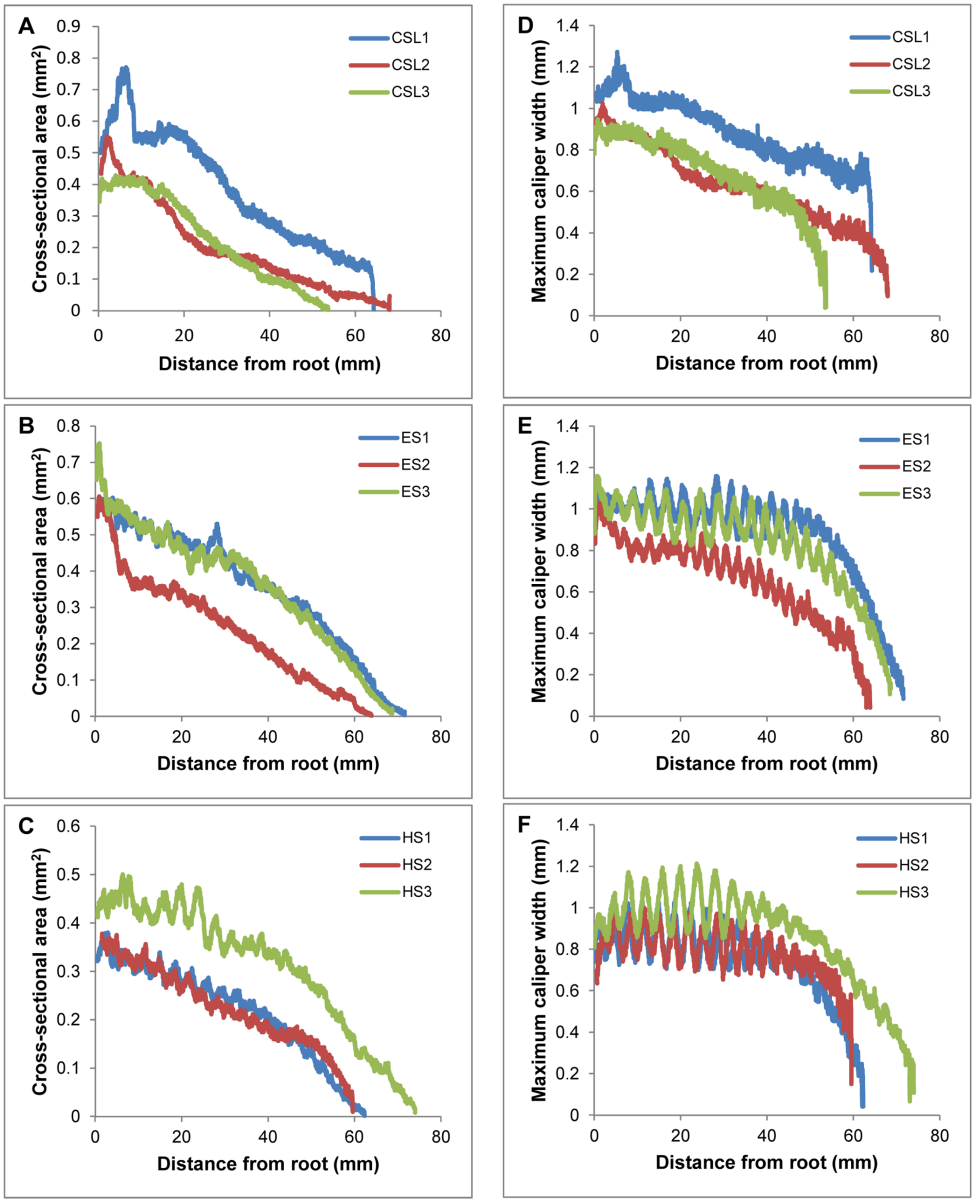
Cross-sectional area and maximum caliper width of vibrissal cross-sectional profiles from CT data. Calculated cross-sectional area along the vibrissal length for three subjects of each species. (A and D) California sea lion; (B and E) elephant seal; (C and F) harbor seal. In all vibrissae, the cross-sectional area gradually decreased from the base of the shaft towards the tip. In undulated vibrissae, the cross-sectional area remained relatively consistent between the crests and troughs, while the maximum caliper width increased and decreased with each undulation.

CT scanning of vibrissal samples allowed for digital cross-sectioning of the hair shafts (Figure 9, S1, S2, S3). Cross-sectional area was tracked along the length of the shaft and maximum caliper width was measured for each cross-section (Figure 10). For all vibrissae, the cross-sectional area gradually decreased from the base of the shaft towards the tip. For the elephant seal and harbor seal vibrissae, the cross-sectional area remained relatively consistent between the crests and troughs, while the maximum caliper width increased and decreased with each undulation. For these undulated vibrissae, although each crest and trough caused the major axes to alternate, the total cross-sectional area was relatively consistent across neighboring undulations.

**Figure 11:**
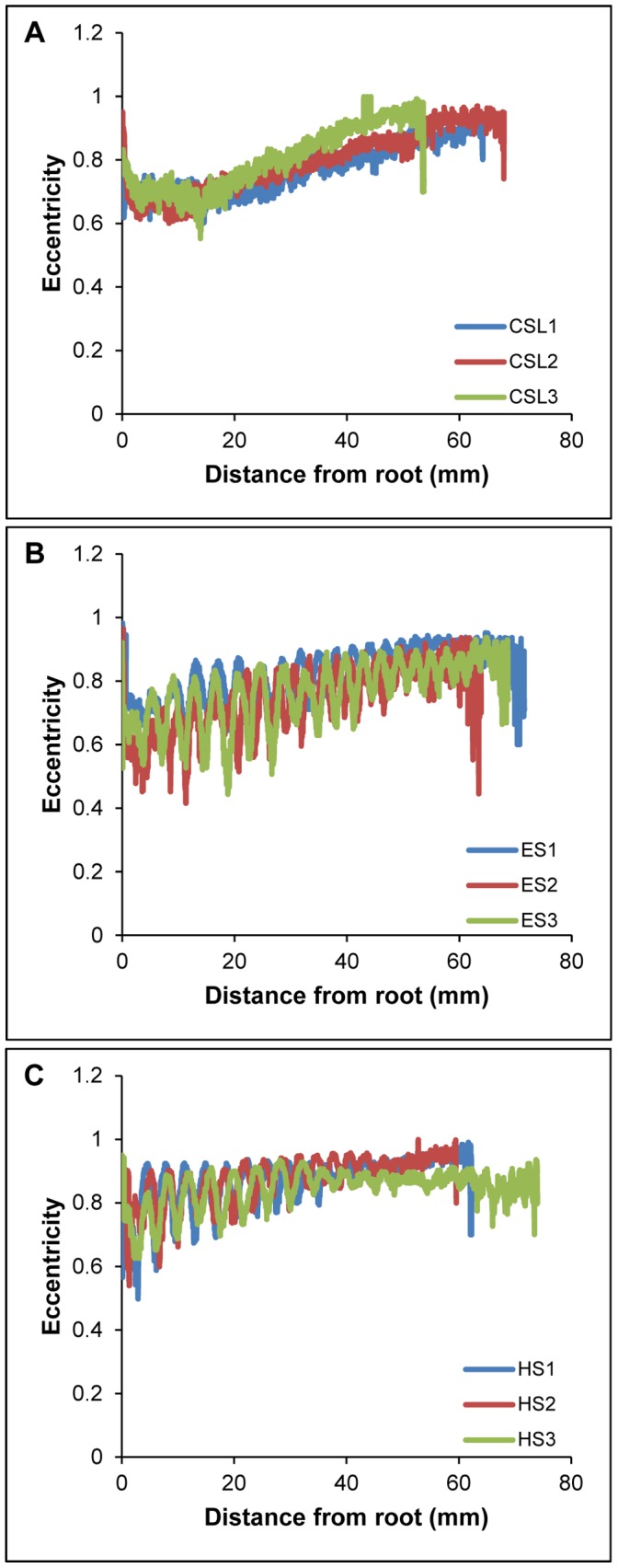
Eccentricity of vibrissal cross-sectional profiles from CT data. Measure of eccentricity, or ellipticity, along the vibrissal length for three subjects of each species. (A) California sea lion; (B) elephant seal; (C) harbor seal. The eccentricity of a perfect circle is 0, while the eccentricity of an ellipse would be >0 but <1. Overall, both smooth and undulated vibrissae show similar degrees of eccentricity. In smooth vibrissae, eccentricity is consistent between neighboring points along the shaft, while in undulated vibrissae eccentricity oscillates with each trough and crest.

**Figure 12:**
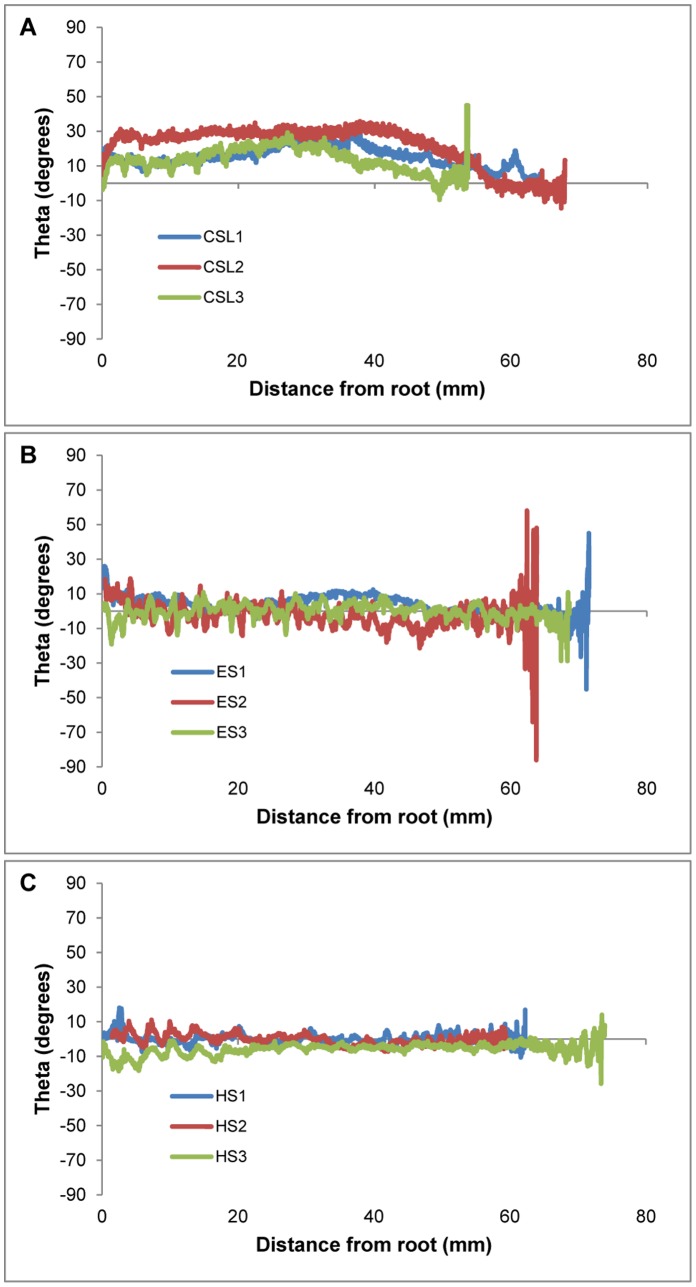
Theta of vibrissal cross-sectional profiles from CT data. Angle of the major axis of the cross-section from horizontal. (A) California sea lion; (B) elephant seal; (C) harbor seal. For smooth vibrissae, theta measurements deviate from zero, while in undulated vibrissae theta measurements centered around zero across the entire length of the vibrissa.

For all vibrissae, the cross-sectional profile became increasingly flattened towards the tip. In addition to this overall trend, local differences in cross-sectional shape were observed between the crest and trough sections of undulated vibrissae. The elephant and harbor seal vibrissae oscillated between more and less compressed ellipsoid cross-sectional shapes from troughs to crests, while the California sea lion vibrissae maintained consistent shape in cross-section between neighboring points along the shaft. These trends can be observed by tracking the measure of eccentricity along the whisker shafts of each individual sample (Figure 11).Theta, the angle of the major axis from horizontal, was calculated for each cross-section (Figure 12). As samples were scanned lying flat against a slide, theta measurements represent the deviation from the axis of the major curvature of the vibrissa. In the undulated vibrissae, theta centered about zero and remained relatively stable from hair base to tip. In contrast, theta in the smooth vibrissae deviated from zero and showed variations along the length of the hair shaft. Theta for these vibrissae ranged from a minimum of −14° to a maximum of 45°. In undulated vibrissae, the orientation of the flattened profile is in-line with the overall curvature of the vibrissa, while in the smooth vibrissae the orientation of the flattened profile is off-axis of the major curvature.

It is difficult to quantify orientation of the vibrissae in live pinnipeds. Underwater photos and high-speed videos of the vibrissal array of a freely swimming harbor seal and California sea lion provide some qualitative comparison between these species. These recordings indicate that differences may occur between species in the direction of vibrissal curvature with respect to the rostro-caudal axis of the body (Figure 5, S4, S5, S6, S7). In footage of the California sea lion swimming with the array protracted, the vibrissae appear to curve ventrally. In contrast, when the harbor seal is swimming with the array protracted, the vibrissae appear straight because the curvature is directed caudally.

## Discussion

When exposed to water flow, both the undulated vibrissae of the seals and the smooth vibrissae of the sea lions showed similar ranges in vibration frequency and velocity. When the vibrissae were tested across a range of orientations, no distinguishing difference was observed as a function of surface structure. Within each angle of orientation, similar values were observed across whisker types. Angle of orientation, rather than species differences and thus surface structure of the vibrissa, had the greatest effect on the frequency and velocity of flow-induced vibrations.

Angle of orientation had a large effect on vibration velocity (Figure 8). For all vibrissal types, peak velocity was lowest at the 0° orientation and corresponded to when the thin edge of the hair was angled into the flow. Rotating the orientation of the vibrissa away from 0° increased the velocity of vibrations along the shaft. In terms of wake tracking, we hypothesize that the 0° orientation would reduce vibrations from forward swimming motion and that the vibrissae would be more sensitive to flow disturbances impinging on the whisker from 90°. It is important to note that flow-disturbances could impinge on the vibrissae from any direction. Self-induced vibrations that could be considered noise to the animal would be minimized, potentially allowing for a greater chance of detecting signals in the water.

While the present study did not find differences between the vibrations of the undulated and smooth vibrissae, previous research by Hanke et al. did find differences between vibrissal types [14]. One possible explanation for this discrepancy is that samples in the previous study were held at one fixed orientation based on the curvature of the vibrissal shaft. If the test positions used in the previous study differed in angling of the flattened profile of the vibrissa into the flow, then more extreme differences could have been observed.

Frequency was also markedly affected by angle of orientation for both the smooth and undulated vibrissae (Figure 8). Measured peak frequency was highest at the 0° orientation, when the thin edge of the hair faced into the flow, and decreased as the vibrissa was rotated to the 90° orientation. This trend is similar to that observed in the calculated frequency of theoretical cylinders that model only the stream-wise diameters of the whiskers and not the surface structure (Table 1). As a vibrissa is rotated from the 0° to the 90° orientation, the broader edge of the vibrissa faces into the flow and the size of the stream-wise diameter increases. This yields higher Reynolds number flow and consequently a lower vibrational frequency. Theoretical values based on stream-wise diameter were within an average of 14% of corresponding measured values for each vibrissa. The similarity in frequency response between measured and theoretical values suggests that a key variable influencing the frequency of vibration is the diameter of the portion of the whisker facing into the flow. This implies that the cross-sectional flattening of the vibrissae, rather than the surface structure, may explain most of the trend observed in measured vibration frequency at the flow speed tested.

CT scanning and digital cross-sectioning of the vibrissae confirmed that this flattening is present in both undulated and smooth vibrissae. Eccentricity calculations revealed that the vibrissae of all species tested were cross-sectionally flattened to some degree and became increasingly flattened toward the distal end (Figure 11). In addition to this overall trend, undulated vibrissae showed oscillating eccentricity values with each crest and trough. Surprisingly, the smooth and undulated vibrissae exhibited similar overall degree of flattening at comparable regions along the vibrissal length, which may be important in reducing self-induced vibration of the sensor. Although it was previously assumed that the undulated vibrissae were more severely compressed in profile [17], these data make it apparent that the smooth vibrissae are also considerably compressed. In addition, the undulated vibrissae showed an interesting compensation along the whisker where the cross-sectional areas of adjacent sections were consistent despite the undulations. Maintaining consistent cross-sectional area might minimize variability in vibrations that would be generated by the hair shaft when exposed to water flow.

Theta, or angle of the major cross-sectional axis from horizontal, was analyzed in order to better understand the relationship between curvature of the vibrissal shaft and the orientation of the cross-sectional profile (Figure 12). For the undulated vibrissae from harbor seals and elephant seals, theta measurements centered around zero along the entire length of the shaft. This indicates that the direction of cross-sectional flattening is in the same plane as the curvature of the hair shaft. In contrast, theta measurements for the smooth vibrissae from sea lions deviated from zero. This indicates that, if the whisker is positioned based on the overall curvature of the hair shaft, the orientation of the flattened profile does not lie directly in this plane. The CT data show that the sea lion whisker would need to be rotated about 10–20 degrees from the plane of the whisker lying flat to have the thinnest edge oriented into the flow.

It is difficult to quantify how these parameters relate to the positioning of the array in a live animal. However, images obtained using high speed recordings provide some qualitative biological framework for understanding these data (Figure 5, S4, S5, S6, S7). These video recordings of a harbor seal and a California sea lion during active swimming allow the overall positioning of the array to be compared between these species. In both subjects, the array remains stable in its protracted position and the vibrissae do not become depressed or swept back as the animal moves through the water. This stable positioning suggests that the vibrissae are able to resist the water pressure from forward movement and we hypothesize that the flattened cross-sectional profile may streamline the vibrissae and aid in the process.

One difference between the harbor seal and the sea lion, which can be seen in the video images, is the curvature of the vibrissae with respect to the rostro-caudal axis of the body. The vibrissae of both species have a distinct major curvature. Comparing the protracted array between these subjects, the vibrissae of the harbor seal appear to be curved caudally, while the vibrissae of the California sea lion appear to be curved ventrally. Taking this into consideration with the theta measurements from CT scanning, it is possible that this positioning may allow the thin edge of the vibrissae to face into the flow. Considering that the laser vibrometer measurements in the flume indicated that the self-induced noise on the vibrissae is minimized at an angle of attack of zero, we can speculate that this array positioning aids in noise reduction. Detailed effort still needs to be made to determine the precise rotational orientation of the array during underwater tracking tasks. However, considering the range of motion of the array in each species, we hypothesize that the 0° condition used in this study is an appropriate generalization of natural orientation.

From the present data, the function of the undulations in pinniped vibrissae is unclear. Under the conditions tested, the undulated surface structure does not appear to minimize self-induced noise on the sensor to a greater degree than vibrissae with a smooth surface. To determine the function of the undulations, subsequent testing should include the introduction of hydrodynamic disturbances and consideration of vibrissal curvature, length, and additional flow speeds. The undulations may provide some advantage in filtering or amplification of a signal when the sensor is exposed to salient hydrodynamic stimuli. Taking into account evidence from previous research using theoretical modeling [14] and behavioral testing [7], [8], pinniped species with undulated vibrissae may be more specialized for hydrodynamic detection. However, the smooth vibrissae should not be considered an inefficient sensor. As can be seen from the comparative laser vibrometer recordings, when oriented at the same angle of orientation, smooth vibrissae exhibit the same capacity as undulated vibrissae to minimize flow-induced vibrations as well as respond strongly to flow in other planes. We hypothesize that this is attributable to the compressed cross-sectional shape of the vibrissal shaft, a shared characteristic between both undulated and smooth vibrissae.

## Supporting Information

Information S1 **CT scan of a California sea lion vibrissa.** A slice-by-slice animation of the CT scan showing successive cross-sections from the vibrissal base to the tip. Slice thickness is 0.03 mm.
(AVI)

Information S2 **CT scan of an elephant seal vibrissa.** A slice-by-slice animation of the CT scan showing successive cross-sections from the vibrissal base to the tip. Slice thickness is 0.03 mm.
(AVI)

Information S3 **CT scan of a harbor seal vibrissa.** A slice-by-slice animation of the CT scan showing successive cross-sections from the vibrissal base to the tip. Slice thickness is 0.03 mm.
(AVI)

Information S4 **Frontal view of a California sea lion vibrissal array.** This view, filmed during active swimming and orientation, shows the array held in the protracted position with the curvature of the vibrissae directed ventrally.
(MP4)

Information S5 **Profile view of a California sea lion vibrissal array.** This view, filmed during active swimming and orientation, shows the array held in the protracted position with the curvature of the vibrissae directed ventrally.
(MP4)

Information S6 **Frontal view of harbor seal vibrissal array.** This view, filmed during active swimming and orientation, shows the array held in the protracted position with the curvature of the vibrissae directed caudally.
(MP4)

Information S7 **Profile view of harbor seal vibrissal array.** This view, filmed during active swimming and orientation, shows the array held in the protracted position with the curvature of the vibrissae directed caudally.
(MP4)
